# A New Color Image Encryption Scheme Using CML and a Fractional-Order Chaotic System

**DOI:** 10.1371/journal.pone.0119660

**Published:** 2015-03-31

**Authors:** Xiangjun Wu, Yang Li, Jürgen Kurths

**Affiliations:** 1 College of Software, Henan University, Kaifeng, China; 2 Potsdam Institute for Climate Impact Research (PIK), Potsdam, Germany; 3 Department of Physics, Humboldt University zu Berlin, Berlin, Germany; Medical University of Graz, AUSTRIA

## Abstract

The chaos-based image cryptosystems have been widely investigated in recent years to provide real-time encryption and transmission. In this paper, a novel color image encryption algorithm by using coupled-map lattices (CML) and a fractional-order chaotic system is proposed to enhance the security and robustness of the encryption algorithms with a permutation-diffusion structure. To make the encryption procedure more confusing and complex, an image division-shuffling process is put forward, where the plain-image is first divided into four sub-images, and then the position of the pixels in the whole image is shuffled. In order to generate initial conditions and parameters of two chaotic systems, a 280-bit long external secret key is employed. The key space analysis, various statistical analysis, information entropy analysis, differential analysis and key sensitivity analysis are introduced to test the security of the new image encryption algorithm. The cryptosystem speed is analyzed and tested as well. Experimental results confirm that, in comparison to other image encryption schemes, the new algorithm has higher security and is fast for practical image encryption. Moreover, an extensive tolerance analysis of some common image processing operations such as noise adding, cropping, JPEG compression, rotation, brightening and darkening, has been performed on the proposed image encryption technique. Corresponding results reveal that the proposed image encryption method has good robustness against some image processing operations and geometric attacks.

## Introduction

Widespread transmission of digital images over various communication media challenges to build credible security methods for the protection of confidential and sensitive information to be transmitted. Hence the security of digital information has become a hot recent topic. Different from text encryption, most conventional ciphers such as Data Encryption Standard (DES), International Data Encryption Algorithm (IDEA), Advanced Encryption Standard (AES), RSA (developed by Rivest, Shamir and Adleman), etc., are not suitable to build cryptosystems for digital images due to inherent features of image data, e.g. bulk data capacity, high redundancy, strong correlation among adjacent pixels, etc. The implementation of these traditional algorithms for image encryption usually requires more computation time and power, and causes also other problems such as in handling various data formatting.

In 1989, Matthews developed the first chaotic stream encryption algorithm [1]. After that, many studies have shown that chaos-based algorithms have advantages in applications of bulk data encryption, because the chaotic signals have cryptographically desirable properties such as extreme sensitivity to initial conditions and parameters, long periodicity, ergodicity, high randomness and mixing [2]. In contrast to traditional cryptographic techniques, it has been found that chaos-based image encryption schemes have superior performance with respect to the trade-offs between the security and efficiency.

Since Fridrich presented a symmetric image encryption algorithm using the two-dimensional standard Baker map in 1998 [3], many image cryptosystems have been developed during the past decades. In practical applications, three types of methods, i.e., permutation, diffusion, and their combined form, are usually employed to design image encryption algorithms [4–9]. The aim of permutation is to transform a meaningful image into a meaningless, disordered and unsystematic image through scrambling the positions of the plain-image pixels, which will enhance the computational complexity of a potential chosen-plaintext attack. In the diffusion process, the values of the original image pixels are changed sequentially so that a tiny change for one pixel can spread out to almost all pixels in the whole image. The image encryption methods using either the permutation-only method or the diffusion-only method have some shortcomings in both security and speed [6, 9].

So far, most image encryption algorithms combine scrambling the pixels positions and modifying the grey values of image pixels to achieve required cryptographic properties. In [10], a new color image encryption method was introduced based on chaotic logistic maps. An external secret key of 80-bit length and two chaotic logistic maps are used. The initial conditions for the logistic maps are obtained using the external secret key. Eight different types of operations are applied to encrypt the image pixels. Wang et al. [11] proposed a new color image encryption algorithm based on the logistic map. Some schemes have been suggested to achieve secure image scrambling based on the Baker map [3], Arnold cat map [12], Standard map [5, 13] etc. Note that the above-mentioned chaos-based image cryptosystems are usually based on the low-dimensional and single chaotic systems, which results in fundamental drawbacks such as insufficient key space, slow speed and weak security function.

In order to improve the security and efficiency performance, many image encryption methods based on three-dimensional chaotic systems, hyperchaos and even spatiotemporal chaos have been presented in recent years [4, 14–23]. For example, Chen et al. [4] generalized a two-dimensional chaotic cat map to a three-dimensional one for constructing a real-time secure symmetric encryption scheme, where the three-dimensional cat map is utilized to create confusion in the relationship between the cipher-image and the plain-image. Mao et al. [14] extended the same idea with the three-dimensional chaotic Baker map. In [15], a digital image encryption scheme based on the mixture of chaotic systems was proposed, where a typical coupled map was mixed with a one-dimensional chaotic map and used for high degree security image encryption. Gao and Chen [16] presented a new image encryption scheme, which used an image total shuffling matrix to shuffle the positions of image pixels and then employed a hyper-chaotic system to confuse the relationship between the plain-image and the cipher-image. The authors in [17] pointed out that this method is very weak to a chosen plain-text attack and a chosen cipher-text attack. In [18], a new image authentication scheme based on a cell neural network with hyper-chaos characteristics (HCCNN) was introduced. Zhu [19] proposed a novel image encryption scheme based on improved hyperchaotic sequences. In this algorithm, the hyperchaotic sequences were firstly modified to generate chaotic key stream that is more suitable for image encryption. Then the final encryption key stream was generated by correlating the chaotic key stream with plain-text. Özkaynak et al. [20] argued that this method is not secure enough, and obtained the secret parameters of the cryptosystem by using chosen plain-text attacks. In [21–23], spatial chaos systems were applied for image encryption in order to overcome the drawbacks of small key space and weak security in widely used one-dimensional chaotic system. However, the previously mentioned algorithms are restricted to grayscale images. Though some of them can be easily extended to handle color images, this extension comes with a cost of substantially increased computation time as a result of additional information required to represent color components.

As is well known, the color images can provide more abundant information than the grayscale ones and are frequently used in many areas. So how to develop a secure encryption algorithm for color images has attracted growing attentions in recent years. Patidar et al. [24] designed a fast loss-less symmetric color image cipher based on the widely used substitution-diffusion architecture which utilized chaotic standard and logistic maps. However, the analysis and simulation results in [25] showed that only a pair of (plain-text/cipher-text) was needed to totally break this cryptosystem. In [26], Huang and Nien proposed a color image cryptosystem using multi-chaotic systems, which is composed of two shuffling stages parameterized by chaotically generated sequences. But this scheme cannot resist known-plaintext attack and chosen-plaintext attack [27]. Rhouma et al. [28] have devised an approach for color image encryption based on one-way coupled-map lattices (OCML). An external secret key of 192-bit length was used to generate the initial conditions and parameters of the OCML by making some algebraic transformations to the secret key. Liu and Wang [29] applied a bit-level permutation and high-dimension chaotic map to encrypt color image. Firstly, a plain color image of size *M* × *N* was converted into a grayscale image of size *M* × 3*N* and the grayscale image was transformed into a binary matrix. Then the matrix was permuted at bit-level by the scrambling mapping generated by a piecewise linear chaotic map (PWLCM). Secondly, the chaotic Chen system was employed to confuse and diffuse the red, green and blue components simultaneously. More recently, several schemes combined DNA computing with chaotic systems to encrypt color images [30, 31]. Such experiments can only be done in a well equipped laboratory using current technology, and it needs higher cost. For these reasons, the studies of DNA cryptography are still focusing on affordable methods in terms of practicality.

Due to the finite precision of digital computers, the most serious defect in single chaotic systems is that the chaotic dynamics degrade fast as they are implemented in computers. Different from this, it has been revealed that spatiotemporal chaotic systems maintain much longer periodicity in digitalization and gain excellent performance in cryptography. On the other hand, chaotic attractors have been discovered in fractional-order systems in the past decade [32–37]. Compared to integer-order systems, the fractional-order systems are found to have more complex dynamics because the fractional derivatives have complex geometrical interpretation due to their nonlocal character and high nonlinearity [38]. In addition, the derivative orders can be also used as secret keys as well, which will increase the key space of the cryptosystem. To our best knowledge, there are few encryption techniques using the fractional-order chaotic systems. Therefore, for the purpose of high security, it is very promising to employ CML and fractional-order chaotic systems in color images encryption.

Motivated by the above discussions, in this paper, we propose a new color image cryptosystem using CML and a fractional-order chaotic system. For the purpose of reaching higher security, higher complexity and higher sensitivity, the present work employs an image division-shuffling process which firstly divides the plain-image into four sub-images, and then shuffles the position of pixels in the whole image. This procedure will significantly enhance the resistance of the proposed cryptosystem against known/chosen-plaintext attacks. In order to increase the security of the presented algorithm, an external secret key of 280-bit length is utilized to generate initial conditions and parameters of the CML and the fractional-order chaotic system by making some algebraic transformations to the key so that the proposed encryption scheme is greatly sensitive to changes in even a single bit of the key. Moreover, to further strengthen the security and sensitivity of the cryptosystem, the CML is used to shuffle the positions of pixels totally, and the fractional-order Chen chaotic system and the plain-image are employed to change the values of the pixels. A simultaneous generation of the key streams and the parallel image division-shuffling process can improve the efficiency of the proposed encryption algorithm. Both theoretical analyses and computer simulations verify the feasibility and superiority of the proposed image cryptosystem. In addition, an extensive tolerance analysis of some common image processing operations such as noise addition, cropping, JPEG compression, rotation, brightening and darkening, is performed on the proposed image encryption technique. The experimental results demonstrate that our method is highly robust against some common image processing operations and geometric attacks.

## The Proposed Chaotic Cryptosystem for Color Images

**Fig 1:**
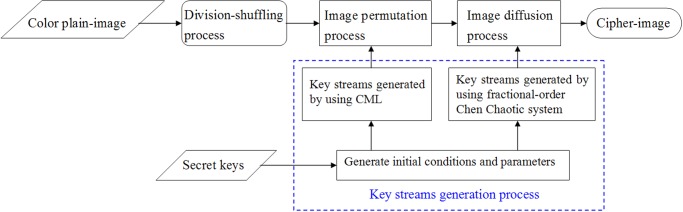
Flowchart of the proposed image encryption algorithm.

The proposed chaos-based cryptosystem for color images consists of the following four parts: i) an image division-shuffling process, ii) a key streams generation process, iii) an image permutation process and iv) an image diffusion process. The flowchart of the image encryption procedure using the proposed scheme is displayed in Fig. 1. Firstly, the plain-image is divided into four sub-images, and then these blocks are shuffled to obtain a disordered image. This process can enhance the resistance of the cipher-image against plaintext attack. Secondly, a 280-bit external secret key is used to generate initial conditions and parameters of the CML and the fractional-order chaotic system. The key streams can be generated by using the obtained initial conditions and parameters to iterate the CML and the fractional-order chaotic system. Thirdly, the positions of the image pixels are permuted by the pseudo-random key stream generated from the CML. In the last stage, the pixel values are modified by the pseudo-random key stream generated from the fractional-order chaotic system. After this, the cipher-image is finally achieved.

### 0.1 The image division-shuffling process

Without loss of generality, we assume that the size of the color plain-image *I* is *M* × *N*, where *M* and *N* are the width and height of the image, respectively. Converting the image *I* into its red, green and blue components, one can get three color matrices *I*_*R*_, *I*_*G*_, *I*_*B*_ with size *M* × *N*. Then combine the red, green and blue matrices horizontally by the following formula (1) and obtain a matrix *I*_1_ with *M* rows and *3N* columns:

$$\left\{ \begin{array}{l} I_{1}(1:M,1:N)=I_{R}(1:M,1:N)\\ I_{1}\left(1:M,(N+1):2N\right)=I_{G}(1:M,1:N)\\ I_{1}\left(1:M,(2N+1):3N\right)=I_{B}(1:M,1:N) \end{array}\right..$$ (1)

**Fig 2:**
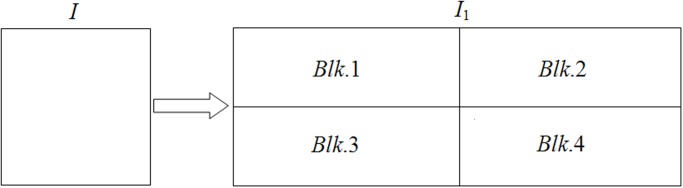
Division of the plain-image.

The image *I*_1_ is decomposed equally into four blocks and each block can be labeled in the form of *Blk*.*k*, *k* = 1, 2, 3, 4, as shown in Fig. 2. The size of each block are given as follows: size(*Blk*. 1) = ⎿*M*/2⏌×⎿3*N*/2⏌, size(*Blk*.2) = ⎿*M*/2⏌×(3*N*−⎿3*N*/2⏌), size(*Blk*.3) = (*M*−⎿*M*/2⏌)×⎿3*N*/2⏌ and size(*Blk*.4) = (*M*−⎿*M*/2⏌)×(3*N*−⎿3*N*/2⏌). If *M* is an odd number, append the first row of *Blk*.4 to the end of *Blk*.2 and delete the first row of *Blk*.4. Then the sizes of blocks *Blk*.2 and *Blk*.4 are obtained as follows: size(*Blk*.2) = (*M*−⎿*M*/2⏌)×(3*N*−⎿3*N*/2⏌) and size(*Blk*.4) = ⎿*M*/2⏌×(3*N*−⎿3*N*/2⏌). And the sizes of the other two blocks keep unchanged. In the following, we will further shuffle the plain-image, which makes the encryption operation more confusing and complex as it adds one extra step to the encryption process.

**Fig 3:**
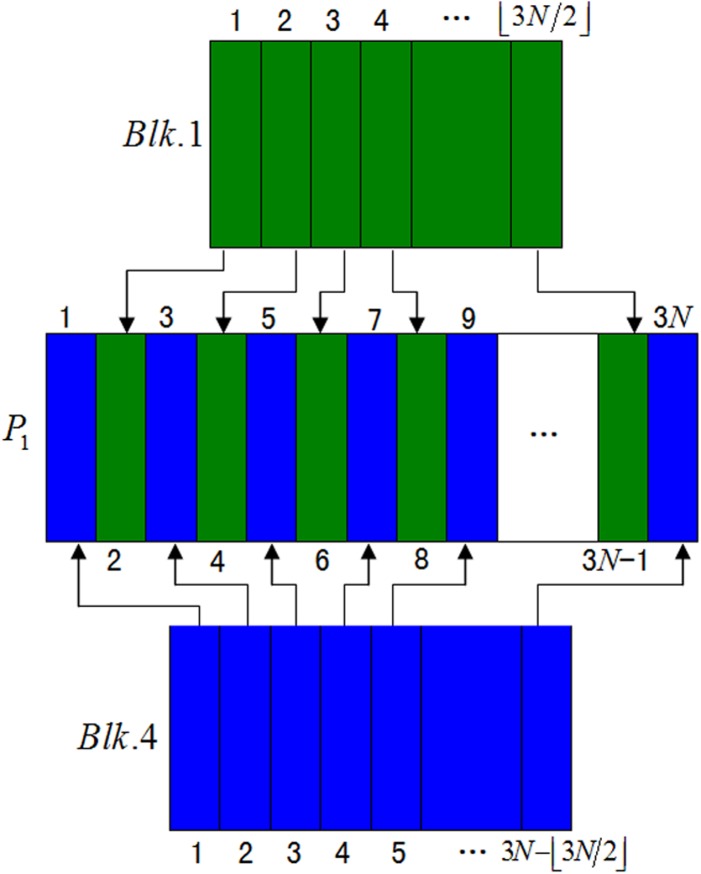
Shuffling of the image blocks *Blk*. 1 and *Blk*. 4.

We firstly create three empty matrices, i.e., *P*_1_ with size ⎿*M*/2⏌×3*N*, *P*_2_ with size (*M*−⎿*M*/2⏌)×3*N* and *I*_2_ with size *M* ×3*N*. Then insert each column of *Blk*.4 in turn into the odd columns of matrix *P*_1_ and insert each column of *Blk*.1 sequentially into the even columns of matrix *P*_1_ as illustrated in Fig. 3. Similarly, insert each column of *Blk*.2 in turn into the odd columns of matrix *P*_2_ and insert each column of *Blk*.3 sequentially into the even columns of matrix *P*_2_. Finally, insert each row of *P*_2_ in turn into the odd rows of matrix *I*_2_ and insert each row of *P*_1_ sequentially into the even rows of matrix *I*_2_. Thus a disordered image matrix *I*_2_ with size *M* ×3*N* is obtained.

Implementing the above procedure, we apply the first complexity to our encryption approach, which significantly enhance the resistance against known/chosen-plaintext attacks.

### 0.2 CML and the fractional-order Chen chaotic system

The cryptosystems based on widely used one-dimensional discrete chaotic maps suffer from fundamental drawbacks such as small key space, slow performance speed and weak security function [39]. To overcome these limitations in the proposed encryption scheme, a 2D coupled map lattice (CML) and the fractional-order Chen chaotic system are utilized to generate the key streams.

**Fig 4:**
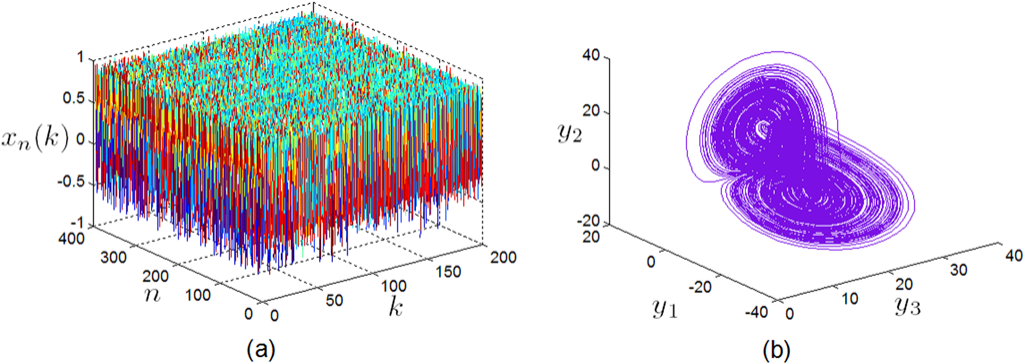
The chaotic behaviors of systems (2) and (3). (a) The spatiotemporal attractor of the 2D CML (2) with *μ* = 1.8 and *ε = 0*.*1*, (b) the chaotic attractor of the fractional-order Chen system (3) with *α*_1_ = *α*_2_ = *α*_3_ = 0.9.

A 2D CML was introduced by Kaneko and Tsuda [40] as a simple model capturing essential features of spatiotemporal dynamics of extended nonlinear systems and later was employed for modeling complex spatial phenomena in diverse areas of science and engineering. Recently, CML was introduced for cryptography of a self-synchronizing stream cipher. The 2D CML is defined as follows:

$$x_{n+1}(k)=(1-\varepsilon )f(x_{n}(k))+\frac{\varepsilon }{2}[f(x_{n}(k-1))+f(x_{n}(k+1))],$$ (2)

where *f*(x) is the mapping function, *n* is the discrete time index, *n* = 0, 1, 2.…,*L*−1, with *L* being the system size, *k* = 1, 2, …,*S* is the lattice site index, and *ε*∈(0, 1) is the coupling constant. In general, periodic boundary conditions *x*_n_(k+L) = *x*_n_(k) are assumed, and *f*(*x*) = 1– *μx* ^2^ is chosen where *μ* is a constant parameter and *μ*∈(0, 2). The chaotic behavior of 2D CML (2) is demonstrated in Fig. 4(a).

Another chaotic system in our scheme is the fractional-order Chen chaotic system, which is described by

$$\left\{ \begin{array}{l} D_{*}^{\alpha_{\text{1}}}y_{\text{1}}=a(y_{\text{2}}-y_{\text{1}})\\ D_{*}^{\alpha_{\text{2}}}y_{\text{2}}=-by_{\text{1}}-y_{\text{1}}y_{\text{3}}+cy_{\text{2}}\\ D_{*}^{\alpha_{\text{3}}}y_{\text{3}}=y_{\text{1}}y_{\text{2}}-dy_{\text{3}} \end{array}\right.,$$ (3)

where *a*, *b*, *c* and *d* are positive system parameters. When *a* = 35, *b* = 7, *c* = 28, *d* = 3, and *α*_j_ ∈[0.8,1] (*j* = 1, 2, 3), the fractional-order Chen system behave chaotically [41], as displayed in Fig. 4(b).

### 0.3 Generation of the initial conditions and parameters

In the proposed scheme, let *L* = *M* × *N* and *S* = 3 for the CML, i.e., *n* = 0, 1, 2,…, *M* × *N* − 1, *k* = 1, 2, 3. In view of the basic need of cryptology, the cipher-text should have a close correlation with the key. There are two ways to accomplish this requirement: one is to mix the key thoroughly into the plain-text through the encryption process, another is to use a good key generation mechanism. Here we use a 280-bit long external secret key (*K*), which is divided into blocks (*K*_*i*_) of 8-bit, to derive the parameters *ε*, *μ*, *a*_*j*_ (*j* = 1, 2, 3) and the initial conditions *x*_0_ (1), *x*_0_ (2), *x*_0_ (3), *y*_1_ (0), *y*_2_ (0) and *y*_3_ (0) in systems (2) and (3). The 280-bit long secret key (*K*) is given by:

$$K=K_{1}K_{\text{2}}K_{\text{3}}\cdots K_{\text{35}}.$$ (4)

The initial conditions and parameters are obtained as follows:

$$x_{0}(1)=\left(\left((K_{1}|K_{3})⊕(K_{\text{5}}|K_{\text{7}})+\sum_{i=1}^{35}K_{i}\right)/\text{257}\right)\mathrm{mod}\;\text{1,}$$ (5)

$$x_{0}(\text{2})=\left(\left((K_{\text{2}}|K_{\text{4}})⊕(K_{\text{6}}|K_{\text{9}})+\sum_{i=1}^{35}K_{i}\right)/\text{257}\right)\mathrm{mod}\;\text{1,}$$ (6)

$$x_{0}(\text{3})=\left(\left((K_{\text{8}}|K_{\text{11}})⊕(K_{\text{13}}|K_{\text{15}})+\sum_{i=1}^{35}K_{i}\right)/\text{257}\right)\mathrm{mod}\;\text{1,}$$ (7)

$$y_{1}(0)=\left(\left((K_{\text{10}}\&K_{\text{12}})⊕(K_{\text{14}}\&K_{\text{16}})+\sum_{i=1}^{35}K_{i}\right)/\text{257}\right)\mathrm{mod}\;\text{3,}$$ (8)

$$y_{\text{2}}(0)=\left(\left((K_{\text{17}}\&K_{\text{19}})⊕(K_{\text{21}}\&K_{\text{23}})+\sum_{i=1}^{35}K_{i}\right)/\text{257}\right)\mathrm{mod}\;\text{4,}$$ (9)

$$y_{\text{3}}(0)=\left(\left((K_{\text{18}}\&K_{\text{20}})⊕(K_{\text{22}}\&K_{\text{24}})+\sum_{i=1}^{35}K_{i}\right)/\text{257}\right)\mathrm{mod}\;\text{ 5,}$$ (10)

$$\varepsilon =\left(\left(\left(K_{\text{25}}⊕K_{\text{27}}⊕K_{\text{29}}+\sum_{i=1}^{35}K_{i}\right)/\text{257}\right)\mathrm{mod}\;\text{1}\right)/\text{10},$$ (11)

$$\mu =\text{1}.\text{8}+\left(\left(\left(K_{\text{26}}⊕K_{\text{28}}⊕K_{\text{30}}+\sum_{i=1}^{35}K_{i}\right)/\text{257}\right)\mathrm{mod}\;\text{1}\right)/\text{10},$$ (12)

$$\alpha_{\text{1}}=\text{0}.\text{92}+\left(\left(\left(K_{\text{31}}⊕K_{\text{33}}⊕K_{\text{35}}+\sum_{i=1}^{35}K_{i}\right)/\text{257}\right)\mathrm{mod}\;\text{1}\right)/\text{15},$$ (13)

$$\alpha_{\text{2}}=\text{0}.\text{92}+\left(\left(\left(K_{\text{30}}⊕K_{\text{32}}⊕K_{\text{34}}+\sum_{i=1}^{35}K_{i}\right)/\text{257}\right)\mathrm{mod}\;\text{1}\right)/\text{15},$$ (14)

$$\alpha_{\text{3}}=\text{0}.\text{92}+\left(\left(\left(K_{\text{10}}⊕K_{\text{15}}⊕K_{\text{25}}+\sum_{i=1}^{35}K_{i}\right)/\text{257}\right)\mathrm{mod}\;\text{1}\right)/\text{15},$$ (15)

where the | operator is the bitwise OR; the & operator is the bitwise AND; ⨁ is the bitwise XOR operator.

Clearly, from Equations (5)–(15), one can see that the initial conditions and parameters of the CML and the fractional-order Chen system are greatly sensitive to changes in even a single bit of the 280-bit secret key. Therefore, the proposed cryptosystem with a key space of 2^280^ can resist any brute-force attack.

### 0.4 Image permutation based on CML

Image data have strong correlations among adjacent pixels in horizontal, vertical, and also diagonal directions for both natural and computer-graphical images. In order to weaken the strong relationship among adjacent pixels, a CML is used to scramble the pixel positions of the image *I*_2_.

To permute the positions of image pixels, we perform the following 11 steps:

**Step 1**. Use the initial conditions *x*_0_(1), *x*_0_(2), *x*_0_(3) and the parameters *ε*, *μ* obtained in Section 0.3 to iterate Equation (2) for *l*_1_ + *MN* times, and discard the former *l*_1_ state values to get rid of harmful effects. One can get three chaotic sequences, i.e., ${\text{D}}_{\text{1}}=\left\{x_{l_{1}+1}(1),x_{l_{1}+2}(1),\cdots ,x_{l_{1}+MN}(1)\right\}$, ${\text{D}}_{\text{2}}=\left\{x_{l_{1}+1}(\text{2}),x_{l_{1}+2}(\text{2}),\cdots ,x_{l_{1}+MN}(\text{2})\right\}$ and ${\text{D}}_{\text{3}}=\left\{x_{l_{1}+1}(\text{3}),x_{l_{1}+2}(\text{3}),\cdots ,x_{l_{1}+MN}(\text{3})\right\}$.

**Step 2**. Preprocess the above three chaotic sequences by the following formula (16):

$${x^{\prime }}_{l_{1}+i}(j)=10^{3}\times x_{l_{1}+i}(j)-\text{Round}(10^{3}\times x_{l_{1}+i}(j)),$$ (16)

where $x_{l_{1}+i}(j)\in {\text{D}}_{j}$, ${x^{\prime }}_{l_{1}+i}(j)\in {{\text{D}}^{\prime }}_{j}$, *i* = 1, 2, …, *MN*, *j* = 1, 2, 3, and Round(*x*) represents the integer function which rounds the real number *x* to the nearest integer. Thus we get three chaotic analogue sequences ${{\text{D}}^{\prime }}_{\text{1}}$, ${{\text{D}}^{\prime }}_{\text{2}}$ and ${{\text{D}}^{\prime }}_{\text{3}}$ with very well expressed random-like properties. Further, transform the vectors ${{\text{D}}^{\prime }}_{j}$ into the matrices MD_*j*_ with size *M* × *N*, where the value in row (**mod**(*i* −1, *M*) + 1) and column ⎾*i*/*M*⏋ of *MD*, denotes the *i*-th value of ${{\text{D}}^{\prime }}_{j}$, i.e., ${\text{MD}}_{j}\left(\left(mod(i-1,M)+\text{1}\right),\left\lceil i/M\right\rceil \right)=x_{l_{1}\;+i}^{'}\;(j)$, *i* = 1, 2, …,*MN*, *j* = 1, 2, 3.

**Step 3**. Divide the image matrix *I*_2_ equally into three matrices from left to right, i.e., *I*_21_ = *I*_2_(1: *M*, 1: *N*), *I*_22_ = *I*_2_(1: *M*, (*N*+1): 2*N*) and *I*_23_ = *I*_2_(1: *M*, (2*N*+1): 3*N*). Transform the matrices *I*_21,_ *I*_22,_ and *I*_23_ into three one-dimensional vectors *VP*_1_, *VP*_2_ and *VP*_3_ with length *MN*, respectively. For example, *VP*_1_ = {*g*_1,1_, *g*_1, 2_, …, *g*_1,*MN*_}^T^ where *g*_1,*i*_ denotes the value of the image pixel in row (**mod**(*i* - 1, *M*) + 1) column ⎾*i*/*M* ⏋of *I*_21_, i.e., *g*_1,*i*_ = *I*_21_((**mod**(*i* - 1, *M*) + 1), ⎾*i*/*M* ⏋), *i* = 1, 2, …,*MN*. Sort *MN* values in *VP*_i_ and attain the sorted vectors $SP_{j}={\left\{{\bar{g}}_{j\text{,}1},{\bar{g}}_{j\text{,}2},\cdots ,{\bar{g}}_{j,MN}\right\}}^{\text{T}}$ (j=1,2,3). Find the positions of values ${\left\{{\bar{g}}_{j\text{,}1},{\bar{g}}_{j\text{,}2},\cdots ,{\bar{g}}_{j,MN}\right\}}^{\text{T}}$ in {*g*_i,1_, *g*_i,2,_…, *g*_i,*MN*_}^T^ and mark the transformation positions *TP*_j_ = {*p*_i,1_, *p*_i,2,_…, *p*_i,*MN*_}^T^ where ${\bar{g}}_{j,i}$ is exactly the value of *g*_*j*,*p*__*ii*_.

**Step 4**. Shuffle the values in *SP*_*j*_ by *TP*_*j*_ (*j* = 1, 2, 3), getting $S{P^{\prime }}_{j}={\left\{{{\bar{g}}^{\prime }}_{j\text{,}1},{{\bar{g}}^{\prime }}_{j\text{,}2},\cdots ,{{\bar{g}}^{\prime }}_{j,MN}\right\}}^{\text{T}}$ where ${{\bar{g}}^{\prime }}_{j,i}={\bar{g}}_{j,p_{j,i}}$. Then transform the three one-dimensional vectors $S{P^{\prime }}_{\text{1}}$, $S{P^{\prime }}_{\text{2}}$ and $S{P^{\prime }}_{\text{3}}$ into the three image matrices *I*_31_, *I*_32_ and *I*_33_ with size *M* × *N*, respectively, where the pixel value of *I*_3,*j*_ in row (**mod**(*i* - 1, *M*) + 1) and column ⎾*i*/*M* ⏋ is equal to the *i*-th value of $S{P^{\prime }}_{j}$, i.e., $I_{3,j}\left(\left(mod(i-1,M)+\text{1}\right),\left\lceil i/M\right\rceil \right)={{\bar{g}}^{\prime }}_{j,i}$, *i* = 1, 2, …, *MN*.

**Step 5**. Repeat Steps 6 to 11 *n* rounds (*n* ≤ min(*M*, *N*)).

**Step 6**. Let *i* ← 1;

**Step 7**. Sort *N* values of the *i*-th row of MD_*j*_ and obtain the transform positions *TM*_*j*,*i*_ = {*pm*_*j*,*i*_(1), *pm*_*j*,*i*_(2),… *pm*_*j*,*i*_(*N*),} (*j* = 1, 2, 3). Scramble the pixel positions of the *i*-th row of image *I*_3,*j*_ according to *TM*_*j*,*i*_, i.e., move the *pm*_*j*,*i*_(1) column of the *i*-th row to the first column, the *pm*_*j*,*i*_(2) column of the *i*-th row to the second column etc., until all columns have been moved; thus a new column transformation of the *i*-th row is generated.

**Step 8**. Let *i* ← *i* + 1, return to Step 7 until *i* reaches *M*. Thus we get three row-permuted matrices *I*_41_, *I*_42_ and *I*_43_.

**Step 9**. Let *i* ← 1;

**Step 10**. Sort *M* values of the *i*-th column of MD_*j*_ and obtain the transform positions *TD*_*j*,*i*_ = {*pd*_*j*,*i*_(1), *pd*_*j*,*i*_(2),…, *pd*_*j*,*i*_(*N*)}^T^ (*j* = 1, 2, 3). Shuffle the pixel positions of the *i*-th column of image *I*_4*j*_ according to *TD*_*j*,*i*_, i.e., move the *pd*_*j*,*i*_(1) row of the *i*-th column to the first row, the *pd*_*j*,*i*_(2) row of the *i*-th column to the second row etc., until all rows have been moved; thus a new row transformation of the *i*-th column is obtained.

**Step 11**. Let *i* ← *i* + 1, return to Step 10 until *i* reaches *N*. Thus we obtain three new total shuffled matrices *I*_51,_ *I*_52_ and *I*_53_, which are respectively the *R*, *G* and *B* color matrices of a new permutation image denoted by *I*_5_.

Obviously, the above permutation-only process just rearranges the pixel positions without changing the pixel’s value. Different from conventional block encryption methods such as DES and AES, the proposed algorithm shuffles the positions of image pixels totally by using the transform positions. Compared with the permutations based on one-dimensional or two-dimensional chaotic maps, the permutation scheme proposed overcomes the drawback of short periodicity, since the relationship between the original and shuffled position of one pixel is not directly related to the chaotic map. However, there are still some potential weak points in these permutation-only algorithms [42], which are weak against statistical attack and known-text attack. To deal with the weakness of pure position permutation method, in the following, a diffusion process is further employed to modify the pixel’s gray value to enhance the security of the encryption algorithm.

### 0.5 Image diffusion based on the fractional-order Chen chaotic system

The encryption algorithm proposed in this paper is based on a permutation-diffusion architecture. In the diffusion stage, the fractional-order Chen chaotic system is employed to generate the key stream for diffusion, and the pixel values are modified sequentially to confuse the relationship between the cipher-image and the plain-image. In some existing chaos-based image ciphers, the key stream used in the diffusion process is solely determined by the key. The same key stream is applied to encrypt different plain-images if the key remains unchanged. An opponent may derive the key stream by the plain-text attack, i.e., by ciphering some special plain-text sequences and then comparing them with the corresponding cipher-text sequences. In order to make the cryptosystem secure against a differential attack, the modification made to a particular pixel depends not only on the corresponding key stream element, but also on the accumulated effect of all previous pixel values. The diffusion process is decomposed into the following 5 steps:

**Step 1**. Arrange the pixels of permuted color matrices *I*_51,_ *I*_52_ and *I*_53_ obtained in Section 0.3 from left to right and then from top to bottom, respectively, we get three one-dimensional vectors *PR* = {*pr*_1_, *pr*_2_,…, *pr*_*MN*_,}, *PG* = {*pg*_1_, *pg*_2_,…, *pg*_*MN*_,} and *PB* = {*pb*_1_, *pb*_2_,…, *pb*_*MN*_,}.

**Step 2**. Use the initial conditions *y*_1_(0), *y*_2_(0), *y*_3_(0), and the fractional orders *α*_*j*_ (*j* = 1, 2, 3) generated in Section 0.3 to iterate Equation (3) for *l*_2_ + *MN* times, and discard the former *l*_2_ state values to avoid transient effects. We then obtain three chaotic sequences, i.e., L_1_ = {*y*_1_(*l*_2_+1), *y*_1_(*l*_2_+2),…, *y*_1_(*l*_2_+*MN*),}, L_2_ = {*y*_2_(*l*_2_+1), *y*_2_(*l*_2_+2),…, *y*_2_(*l*_2_+*MN*),} and L_3_ = {*y*_3_(*l*_2_+1), *y*_3_(*l*_2_+2),…, *y*_3_(*l*_2_+*MN*)}.

**Step 3**. To get the key streams, preprocess the above three chaotic sequences according to the following formula (17):

$${y^{\prime }}_{j}(l_{2}+i)=\text{Round}\left(\text{Abs}\left(y_{j}(l_{2}+i)-\text{Fix}\left(y_{j}(l_{2}+i)\right)\right)\right)\times 10^{\text{14}}\text{mod 256,}$$ (17)

where *y*_j_(*l*_2_ + *i*) ∊ L_*j*_, $y_{j}^{'}(l_{2}+i)\in \;{\text{L}}_{j}^{'}$, *i* = 1, 2, …, *MN*, *j* = 1, 2, 3, and Round(*x*) represents the integer function which has the same meaning as that in Equation (16), the function Abs(*x*) returns the absolute value of *x* and the function Fix(*x*) returns the value of *x* to the nearest integer towards zero. Thus we obtain three key streams ${\text{L}}_{1}^{'}$, ${\text{L}}_{2}^{'}$ and ${\text{L}}_{3}^{'}$.

**Step 4**. Calculate the corresponding pixel data of the cipher-image by using the values of the currently operated pixel and the previously operated pixels, according to the following formula:

$$C\_R(i)=\left(\left(PR(i)⊕{\text{L}}_{1}^{'}(i)\right)⊕\left(C\_R(i-\text{1})⊕{\text{L}}_{1}^{'}(i-\text{1})\right)\right)⊕\left(C\_G(i-\text{1})⊕C\_B(i-\text{1})\right),$$ (18)

$$C\_G(i)=\left(\left(PG(i)⊕{{\text{L}}^{\prime }}_{\text{2}}(i)\right)⊕\left(C\_G(i-\text{1})⊕{{\text{L}}^{\prime }}_{\text{2}}(i-\text{1})\right)\right)⊕\left(C\_R(i-\text{1})⊕C\_B(i-\text{1})\right),$$ (19)

$$C\_B(i)=\left(\left(PB(i)⊕{{\text{L}}^{\prime }}_{\text{3}}(i)\right)⊕\left(C\_B(i-\text{1})⊕{{\text{L}}^{\prime }}_{\text{3}}(i-\text{1})\right)\right)⊕\left(C\_R(i-\text{1})⊕C\_G(i-\text{1})\right),$$ (20)

where *i* = 1, 2, …, *MN*, ⨁ is the bitwise XOR operator, *PR*(*i*), *PG*(*i*) and *PB*(*i*) are the current plain pixel values, *C_R*(*i*), *C_G*(*i*) and *C_B*(*i*) are the current cipher pixel values, ${{\text{L}}^{\prime }}_{\text{1}}(i)$, ${{\text{L}}^{\prime }}_{\text{2}}(i)$ and ${{\text{L}}^{\prime }}_{\text{3}}(i)$ are the current key stream elements, *C_R*(*i − 1*), *C_G*(*i − 1*) and *C_B*(*i − 1*) are the previous cipher pixel values, and ${{\text{L}}^{\prime }}_{\text{1}}(i-\text{1})$, ${{\text{L}}^{\prime }}_{\text{2}}(i-\text{1})$ and ${{\text{L}}^{\prime }}_{\text{3}}(i-\text{1})$ are the previous key stream elements. The first cipher pixel values *C_R*(*1*), *C_G*(*1*) and *C_B*(*1*) are set as follows:

$$C\_R(\text{1})=\left(\left(PR(\text{1})⊕{{\text{L}}^{\prime }}_{\text{1}}(\text{1})\right)⊕\left(PR(MN)⊕{{\text{L}}^{\prime }}_{\text{1}}(MN)\right)\right)⊕\left(PG(MN)⊕PB(MN)\right),$$ (21)

$$C\_G(\text{1})=\left(\left(PG(\text{1})⊕{{\text{L}}^{\prime }}_{\text{2}}(\text{1})\right)⊕\left(PG(MN)⊕{{\text{L}}^{\prime }}_{\text{2}}(MN)\right)\right)⊕\left(PR(MN)⊕PB(MN)\right),$$ (22)

$$C\_B(\text{1})=\left(\left(PB(\text{1})⊕{{\text{L}}^{\prime }}_{\text{3}}(\text{1})\right)⊕\left(PB(MN)⊕{{\text{L}}^{\prime }}_{\text{3}}(MN)\right)\right)⊕\left(PR(MN)⊕PG(MN)\right).$$ (23)

**Step 5**. Transform three encrypted vectors *C_R*, *C_G* and *C_B* with length *MN* into three matrices with size *M* × *N*, i.e., *CR*, *CG* and *CB*, respectively, which are the *R*, *G*, *B* components of the ciphered image *C*. Thus we finally obtain the encrypted image.

Since the CML and the fractional-order chaotic system have a nonlinear structure and more complex dynamics than low-dimensional ones, the proposed chaos-based cryptosystem is greatly sensitive to a change in even a single bit of the 280-bit long secret key. Moreover, using the division-shuffling process and the permutation-diffusion process, a slight change of plain-image pixel causes a significant change in the cipher-image, which makes a differential analysis inefficient and practically useless. These features will in turn strengthen the security and sensitivity of the cryptosystem. In addition, the generation of pseudo-random numbers by the CML and the fractional-order Chen chaotic system and the division-shuffling process on the plain-image can be carried out simultaneously, i.e., in a parallel manner, which promotes the speed performance of the proposed image encryption algorithm. Therefore, our proposed scheme has higher security and overcomes the limitations in the image cryptosystem based on one-dimensional or two-dimensional chaotic maps.

### 0.6 Design of image decryption algorithm

Because the presented color image encryption algorithm is a symmetric cryptosystem, the decryption procedure is similar to that of the encryption process but just in the reversed order. However, some remarks should be considered in the decryption process, which are summarized as follows:

**Remark 1**. We can rewrite Equations (18)–(23) to give the pixel values in the RGB components:

$$PR(i)=\left(\left(C\_R(i)⊕{{\text{L}}^{\prime }}_{\text{1}}(i)\right)⊕\left(C\_R(i-\text{1})⊕{{\text{L}}^{\prime }}_{\text{1}}(i-\text{1})\right)\right)⊕\left(C\_G(i-\text{1})⊕C\_B(i-\text{1})\right),$$ (24)

$$PG(i)=\left(\left(C\_G(i)⊕{{\text{L}}^{\prime }}_{\text{2}}(i)\right)⊕\left(C\_G(i-\text{1})⊕{{\text{L}}^{\prime }}_{\text{2}}(i-\text{1})\right)\right)⊕\left(C\_R(i-\text{1})⊕C\_B(i-\text{1})\right),$$ (25)

$$PB(i)=\left(\left(C\_B(i)⊕{{\text{L}}^{\prime }}_{\text{3}}(i)\right)⊕\left(C\_B(i-\text{1})⊕{{\text{L}}^{\prime }}_{\text{3}}(i-\text{1})\right)\right)⊕\left(C\_R(i-\text{1})⊕C\_G(i-\text{1})\right),$$ (26)

$$PR(\text{1})=\left(\left(C\_R(\text{1})⊕{{\text{L}}^{\prime }}_{\text{1}}(\text{1})\right)⊕\left(PR(MN)⊕{{\text{L}}^{\prime }}_{\text{1}}(MN)\right)\right)⊕\left(PG(MN)⊕PB(MN)\right),$$ (27)

$$PG(\text{1})=\left(\left(C\_G(\text{1})⊕{{\text{L}}^{\prime }}_{\text{2}}(\text{1})\right)⊕\left(PG(MN)⊕{{\text{L}}^{\prime }}_{\text{2}}(MN)\right)\right)⊕\left(PR(MN)⊕PB(MN)\right),$$ (28)

$$PB(\text{1})=\left(\left(C\_B(\text{1})⊕{{\text{L}}^{\prime }}_{\text{3}}(\text{1})\right)⊕\left(PB(MN)⊕{{\text{L}}^{\prime }}_{\text{3}}(MN)\right)\right)⊕\left(PR(MN)⊕PG(MN)\right).$$ (29)

where *i* = *MN*,(*MN − 1*), (*MN − 2*),…,3,2.

**Remark 2**. Perform the reverse operations to remove the effect of permutation. All operations are the same as steps 3–11 in the image permutation process.

**Remark 3**. Use the same method in the image division-shuffling process but in the reversed order to recover the original color image.

**Remark 4**. Note that since the decryption process requires the same key streams for decrypting the cipher-image, the same 280-bit long external secret key *K* = *K*_1_ *K*_2_ *K*_3_…*K*_35_ should be applied for decryption. Hence, according to Section 0.2, it is possible to set the same initial conditions *x*_0_(1), *x*_0_(2), *x*_0_(3), *y*_1_(0), *y*_2_(0), and *y*_3_(0), and the parameters *ε*, *μ*, *α*_*j*_ (*j* = 1, 2, 3).

**Fig 5:**
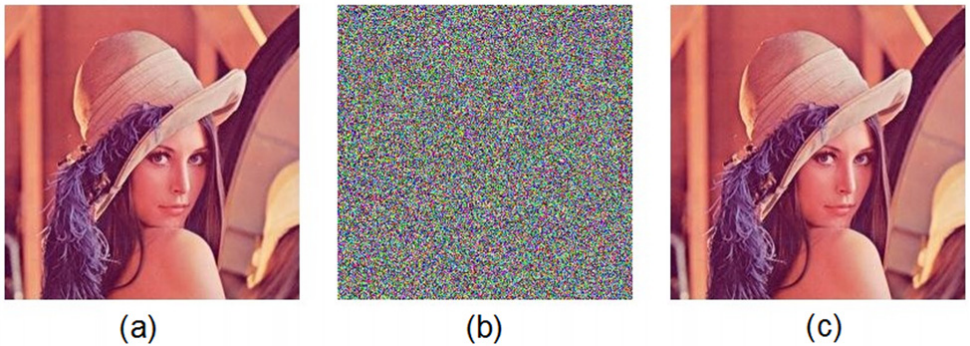
Experimental results for Lena image. (a) Original image of Lena, (b) encrypted image of Lena, (c) decrypted image of Lena.

**Fig 6:**
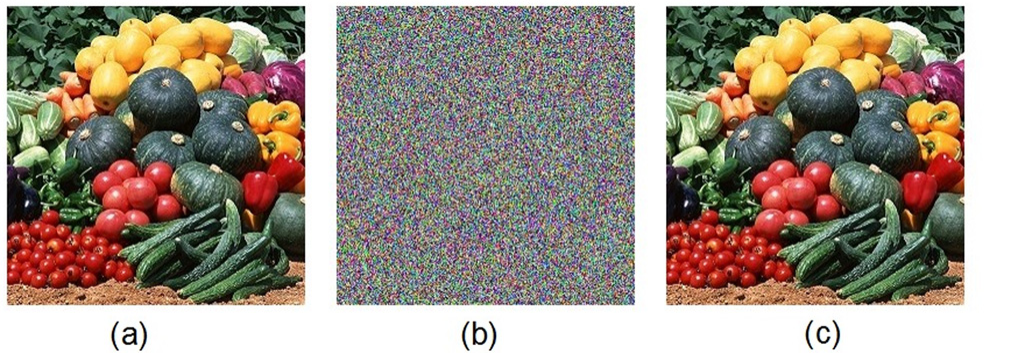
Experimental results for Vegetables image. (a) Original image of Vegetables, (b) encrypted image of Vegetables, (c) decrypted image of Vegetables.

Figs. 5 and 6 show the encryption and decryption of two color images of Lena and Vegetables with size 256 × 256, respectively, where the secret key is chosen as “2eea2814e6087660406d59f82f740bfd9c2e5e463d96bdafe482c8054f457bab5cd180” (in hexadecimal). Throughout this paper, the original image of Lena is freely available at the USC-SIPI image database [43].

## Performance and Security Analysis

In this section, the performance of the proposed image cryptosystem is analyzed by using different security test measures. These measures are taken as follows: key space analysis, statistical analysis including histogram analysis and computing the correlation coefficients of adjacent pixels, information entropy analysis, test security against differential attack including calculating the number of pixel change rate (NPCR) and unified average changing intensity (UACI), and key sensitivity analysis.

### 1.1 Key space analysis

The size of the key space is the total number of different keys that can be applied in the encryption/decryption process. The key space should be large enough to make brute-force attacks infeasible. From the cryptographic point of view, the size of the key space should not be smaller than 2^100^ to ensure a high level of security [44]. Since the secret key of the proposed scheme is 280-bit long, the key space is 2^280^, which is sufficiently large enough to resist a brute-force attack.

### 1.2 Statistical analysis

Shannon suggested that diffusion and confusion should be employed in a cryptosystem [8] for the purpose of frustrating a powerful statistical analysis. An ideal cipher should be robust against any statistical attack. In order to demonstrate the robustness of the proposed image encryption scheme, we have performed some statistical tests on the histograms of the ciphered images and on the correlations of adjacent pixels in the ciphered image.

#### A. Histogram analysis

Image histogram is a significant feature in image analysis. Indeed, one can see the frequency of each gray level from the histograms, which can leak image information. For a good encryption algorithm, the distribution of cipher-text should hide the redundancy of plain-text and not leak any information about the plain-text or the relationship between the plain-text and the cipher-text.

**Fig 7:**
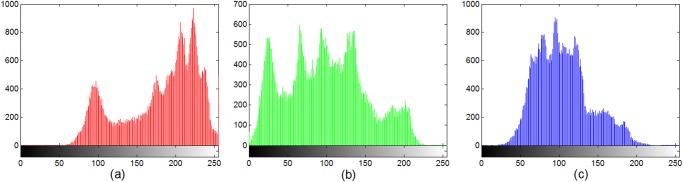
Histogram of the original image of Lena in the (a) red, (b) green, (c) blue, components.

**Fig 8:**
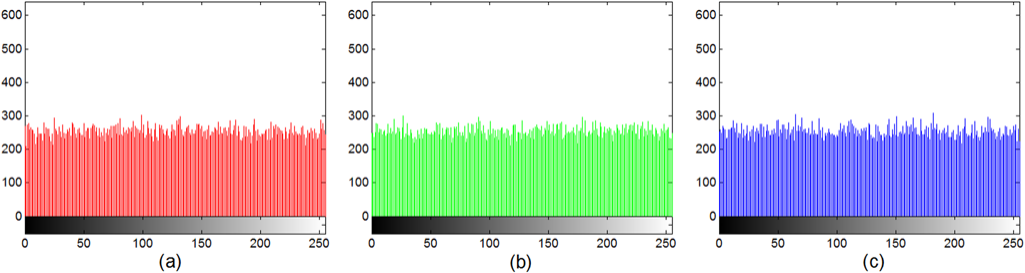
Histogram of the encrypted image of Lena in the (a) red, (b) green, (c) blue, components.

Figs. 7 and 8 display the histograms of the color plain-image “Lena” (Fig. 5(a)) and the corresponding cipher-image (Fig. 5(b)), respectively. From these figures, one can clearly see that the histograms of the encrypted image are fairly uniform and significantly different from those of the plain-image. The statistical feature of the plain-image is enhanced in such a manner that the cipher-image has a uniform gray level distribution and good balance property. Hence it does not offer any clue to be used in a statistical analysis attack on the encrypted image.

#### B. Correlation analysis of two adjacent pixels

It is well known that the adjacent pixels of the plain images are highly correlated either in horizontal, vertical or diagonal directions. An efficient image encryption algorithm should decrease the correlation of two adjacent pixels in the ciphered image as low as possible.

In the following, the correlation between all pairs of two horizontally adjacent pixels, all pairs of two vertically adjacent pixels, and 2000 pairs randomly selected of two diagonally adjacent pixels in the plain-image and cipher-image is tested. The correlation coefficients between two adjacent pixels in an image are calculated using the following equations:

$$r_{xy}=\frac{\mathrm{cov}(x,y)}{\sqrt{D(x)D(y)}},$$ (30)

$$E(x)=\frac{1}{N}\sum_{i=1}^{N}x_{i},$$ (31)

$$D(x)=\frac{1}{N}\sum_{i=1}^{N}{\left(x_{i}-E(x)\right)}^{2},$$ (32)

$$\mathrm{cov}(x,y)=\frac{1}{N}\sum_{i=1}^{N}\left(x_{i}-E(x))(y_{i}-E(y)\right),$$ (33)

where *x* and *y* are gray level values of two adjacent pixels in the image, *N* is the total number of pixels selected from the image, *E*(*x*) and *E*(*y*) are the mean values of *x*_*i*_ and *y*_*i*_, respectively.

**Fig 9:**
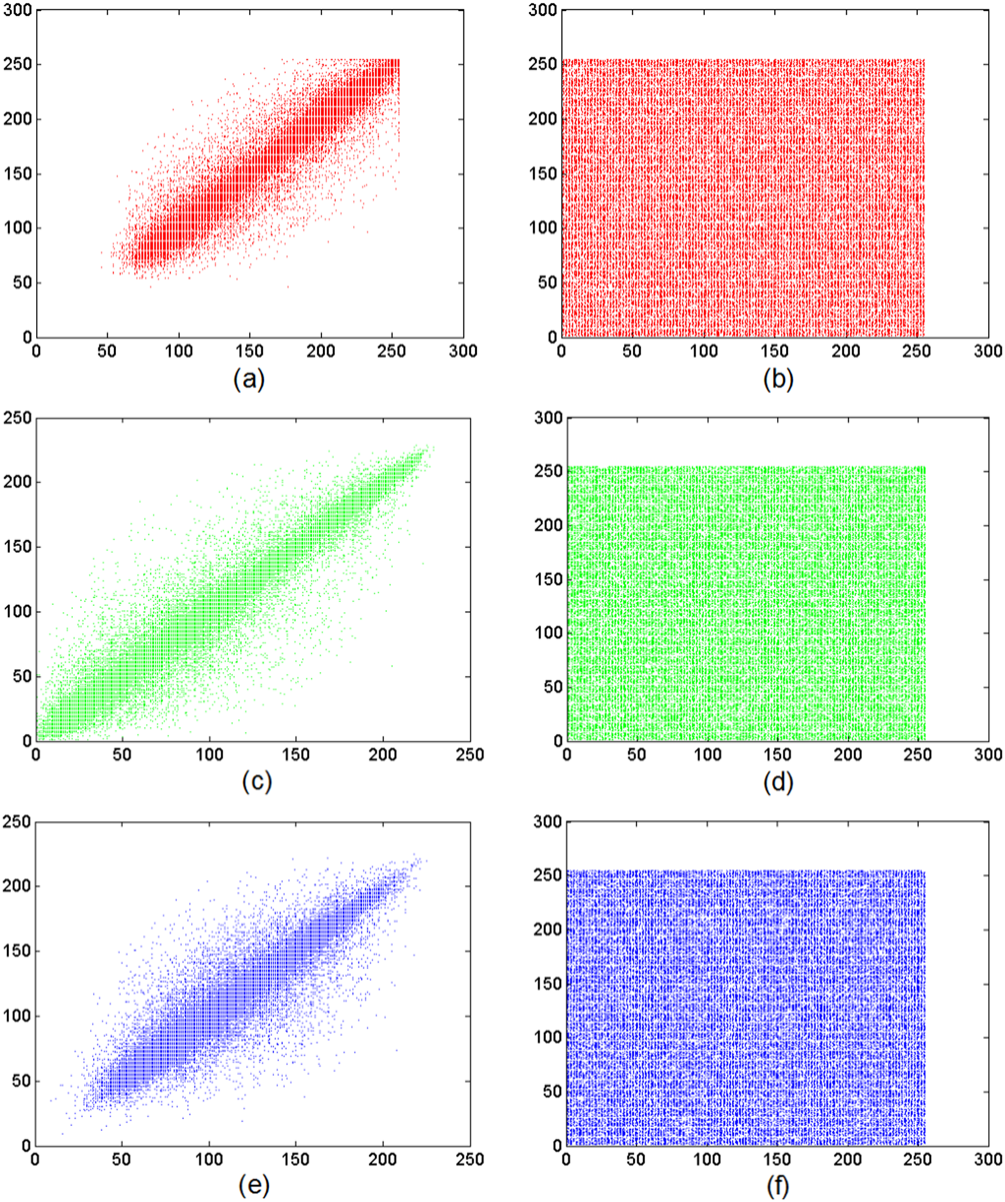
Vertical direction correlations of two adjacent pixels. Frames (a), (c) and (e) show the distribution of two vertically adjacent pixels in the plain-image of Lena in the (a) red, (b) green and (c) blue components, respectively. Frames (b), (d) and (f) display the distribution of two vertically adjacent pixels in the encrypted image of Lena in the (b) red, (d) green and (f) blue components, respectively.

Fig. 9 shows the vertical relevance of adjacent pixels in the plain-image of Lena (Fig. 5(a)) and the encrypted one (Fig. 5(b)). The detailed results of the correlation coefficients for two horizontally (vertically and diagonally) adjacent pixels in the red, green and blue components of the original plain-image and the encrypted one are given in Table 1. These results clearly show that the correlation coefficients of the plain-image are close to 1, while those of the cipher-image are nearly 0 and the distribution of adjacent pixels is fairly uniform. It indicates that the proposed algorithm has successfully reduced the correlation of adjacent pixels in the plain-image so that neighboring pixels in the cipher-image virtually have no correlation. So the proposed algorithm can resist statistical attacks. Furthermore, the comparison performed in Table 2 demonstrates that the proposed scheme in this paper is superior to other methods reported in the literature. The cipher-image using our proposed algorithm has the highest performance in the horizontal, vertical and diagonal directions.

**Table 1:** Correlation coefficients of two adjacent pixels in the plain-image and cipher-image.

Correlation direction	Plain-image			Ciphered-image		
	Red	Green	Blue	Red	Green	Blue
Horizontal	0.94003	0.94082	0.89333	0.00238	-0.00563	-0.00780
Vertical	0.96795	0.97099	0.94265	0.00098	-0.00368	0.00312
Diagonal	0.88295	0.86469	0.74511	-0.01475	-0.02953	-0.02467

**Table 2:** Comparison of correlation coefficients of two adjacent pixels in different directions using the proposed algorithm with some other algorithms.

	Correlation direction		
	Horizontal	Vertical	Diagonal
The original Lena image	0.92473	0.96053	0.83092
The proposed algorithm	-0.00368	0.00014	-0.02298
Ref. [26]	0.1257	0.0581	0.0504
Ref. [28]	0.0681	0.0845	-
Ref. [30]	0.0042	0.0033	0.0024
Ref. [37]	-0.00124	0.00176	0.00193
Ref. [45]	-0.0018	0.00033	0.00427

### 1.3 Information entropy analysis

There are various kinds of entropy, such as K-S entropy, K2 entropy, Tsallis entropy and information entropy *etc*. Information entropy, also called Shannon entropy, which was introduced by Shannon in 1948, is described by

$$H(s)=\sum_{i=0}^{2^{N}-1}p(s_{i})\log_{\text{2}}\frac{\text{1}}{p(s_{i})},$$ (34)

where *s* denotes the information source, *N* is the number of bits to represent a symbol *s*_*i*_ ∊ *s*, *p*(*s*_*i*_) is the probability of symbol *s*_*i*_. For a purely random source emitting 2^*N*^ symbols, i.e., *s* = 2^*N*^, the information entropy is *H*(*s*) = *N*. In fact, a practical information source seldom generates random messages. Therefore, in general its entropy value is smaller than the maximum one. However, when the messages are encrypted, their entropy should ideally be *N*. If the output of such a cipher emits symbols with entropy less than *N*, there is a certain degree of predictability, which threatens its security. In order to design a good cryptosystem, the information entropy of the cipher-image should be as close to the ideal case as possible.

For the cipher-image (Fig. 5(b)) of the original Lena image (Fig. 5(a)) encrypted using the proposed scheme, we record the number of occurrence of each cipher-image pixel *m*_*i*_ and calculate the probability of occurrence for the red, green and blue components of the cipher-image, respectively. The entropy of the three color components of the cipher-image is:

$$H_{R}(m)=\sum_{i=0}^{2^{\text{8}}-1}p(R_{i})\log_{\text{2}}\frac{\text{1}}{p(R_{i})}=\text{7}.\text{9893}\approx \text{8},$$

$$H_{G}(m)=\sum_{i=0}^{2^{\text{8}}-1}p(G_{i})\log_{\text{2}}\frac{\text{1}}{p(G_{i})}=\text{7}.\text{9898}\approx \text{8},$$

$$H_{B}(m)=\sum_{i=0}^{2^{\text{8}}-1}p(B_{i})\log_{\text{2}}\frac{\text{1}}{p(B_{i})}=\text{7}.\text{9894}\approx \text{8},$$

where *R*_*i*,_ *G*_*i*_ and *B*_*i*_ are the color components of the pixel *m*_*i*_. The values obtained are very close to the theoretical maximum value *N* = 8 for the three color components, which indicates that information leakage in the encryption process is negligible and the cryptosystem is secure against an entropy attack. Further, Table 3 compares information entropy using the proposed algorithm with those using the existing algorithms mentioned in Refs. [29, 46]. Obviously, the entropy obtained using our proposed algorithm is indeed closer to the maximum.

**Table 3:** Comparison of the information entropy using the proposed algorithm with some other algorithms.

Algorithm	Entropy		
	Red	Green	Blue
The proposed algorithm	7.9893	7.9898	7.9894
Ref. [29]	7.9871	7.9881	7.9878
Ref. [46]	7.9278	7.9744	7.9705

### 1.4 Differential attack

In general, an opponent may make a slight change (e.g., modify only one pixel) in the plain-image and compare the ciphered images to find out some meaningful relationship between the plain-image and the cipher-image, which can facilitate in determining the secret key. If one minor change in the plain-image can cause a significant change in the cipher-image, with respect to diffusion and confusion, then this differential attack would become very inefficient and practically useless.

As a general requirement for all the image encryption schemes, the encrypted image should be clearly different from its original form. Such a difference can be measured by means of two criteria, namely, the number of pixel change rate (NPCR) and the unified average changing intensity (UACI) [4, 28]. The more NPCR approaches 100%, the more effective for the cryptosystem to resist a plain-text attack. The larger UACI is, the more effective for the cryptosystem to resist a differential attack.

The formulas for calculating NPCR and UACI are described, respectively, as follows:

$${\text{NPCR}}_{R,G,B}=\frac{\sum_{i,j}D_{R,G,B}(i,j)}{M\times N}\times 100\%,$$ (35)

$${\text{UACI}}_{R,G,B}=\frac{\sum_{i,j}\frac{\left|C_{R,G,B}(i,j)-{C^{\prime }}_{R,G,B}(i,j)\right|}{255}}{M\times N}\times 100\%,$$ (36)

where *M* and *N* are the width and height of the image, *C*_*R*,*G*,*B*_ and ${C^{\prime }}_{R,G,B}$ are the two encrypted images before and after only one pixel of the plain-image is changed, respectively, *C*_*R*,*G*,*B*_(*i*, *j*) and ${C^{\prime }}_{R,G,B}(i,j)$ are the values of the corresponding red, green or blue component in the two cipher-images, respectively. The matrix *D*_*R*,*G*,*B*_ is defined as follows: if $C_{R,G,B}(i,j)={C^{\prime }}_{R,G,B}(i,j)$, then *D*_*R*,*G*,*B*_ *(i*, *j)* = 0; otherwise, *D*_*R*,*G*,*B*_ *(i*, *j)* = 1. For instance, for two random images with 256 × 256 pixels and 24-bit true color, the expected values of NPCR_*R*,*G*,*B*_ and UACI_*R*,*G*,*B*_ are, respectively, computed as follows: NPCR_*R*_ = NPCR_*G*_ = NPCR_*B*_ = 99.6094% and UACI_*R*_ = UACI_*G*_ = UACI_*B*_ = 33.4635%.

**Fig 10:**
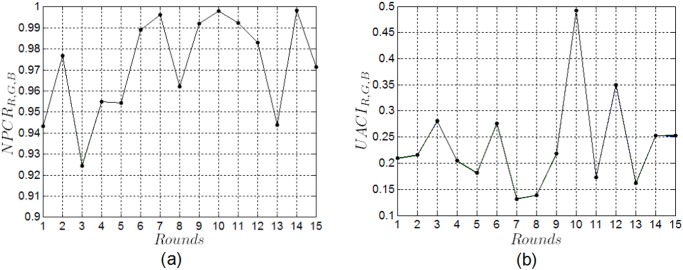
NPCR and UACI performance analysis of the proposed scheme. (a) NPCR versus permutation rounds, (b) UACI versus permutation rounds.

To test the NPCR and UACI of the proposed cryptosystem, two plain images with only one bit difference are employed, i.e., the original color image of Lena, and the other one which is obtained by randomly modifying the value ‘213’ of the pixel located at (2,129) of the red component in the original image as ‘214’. Their corresponding ciphered images are obtained by encrypting the two plain images with the same key after *n* rounds of the proposed permutation process. The NPCR_*R*,*G*,*B*_ and UACI_*R*,*G*,*B*_ versus permutation rounds *n* are plotted in Fig. 10(a) and (b), respectively. One can find that different NPCR_*R*,*G*,*B*_ and UACI_*R*,*G*,*B*_ are obtained after different permutation rounds; after *n* = 14 permutation rounds, we get the largest value of NPCR_*R*,*G*,*B*_, while the value of UACI_*R*,*G*,*B*_ is small; after *n* = 10 permutation rounds, we obtain the largest values of UACI_*R*,*G*,*B*_, and the values of NPCR_*R*,*G*,*B*_ are more than 99.7%. From Fig. 10, we also find that the performance of NPCR_*R*,*G*,*B*_ and UACI_*R*,*G*,*B*_ are better after *n* = 10 permutation rounds than that obtained after other permutation rounds. The detailed results of NPCR_*R*,*G*,*B*_ and UACI_*R*,*G*,*B*_ with permutation rounds *n* = 10 are given in Table 4. One can easily see that the NPCR_*R*,*G*,*B*_ is over 99.79% and the UACI_*R*,*G*,*B*_ is over 49.19%, which implies that the proposed image cryptosystem is very sensitive to tiny changes in the plain-image. A slight change in the original image will result in a significant change in the ciphered image, so the proposed scheme can well resist a known/chosen plaintext attack.

**Table 4:** NPCR and UACI of the color image of Lena when changing one pixel and permutation rounds *n* = 10.

	NPCR (%)	UACI (%)
Red	99.7909	49.1964
Green	99.7925	49.2234
Blue	99.7910	49.2374

Table 5 compares the NPCR_*R*,*G*,*B*_ and UACI_*R*,*G*,*B*_ for the proposed method and the schemes in Refs. [11, 26, 28, 30, 37, 45, 46] on the color image of Lena. As it is clear from the simulation results, our proposed cryptosystem achieves a higher performance by having NPCR_*R*,*G*,*B*_ ⩾ 99.79% and UACI_*R*,*G*,*B*_ ⩾ 49.19%.

**Table 5:** Comparison of the average NPCR and UACI values on the color image of Lena.

Algorithm	Average NPCR (%)	Average UACI (%)
The proposed scheme	99.7915	49.2191
Ref. [11]	99.6358	33.4428
Ref. [26]	99.52	26.7933
Ref. [28]	99.5843	33.3755
Ref. [30]	99.2173	33.4055
Ref. [37]	42.7519	13.2874
Ref. [45]	99.9654	33.5720
Ref. [46]	99.6062	33.8981

### 1.5 Key sensitivity analysis

An efficient encryption scheme has also to be sensitive to the secret key, i.e., a very small change in the key will cause a significant change in the output. Suppose that two 280-bit secret keys are chosen randomly as: K1 =“ 2eea2814e6087660406d59f82f740bfd9c2e5e463d96bdafe482c8054f457bab5cd180” and K2 =“ 2eea2814e6087660406d59f82f740bfd9c2e5e463d96bdafe482c8054f457bab5cd181”. Obviously, two keys are different in only one bit. The key sensitivity test is carried out as follows.

**Fig 11:**
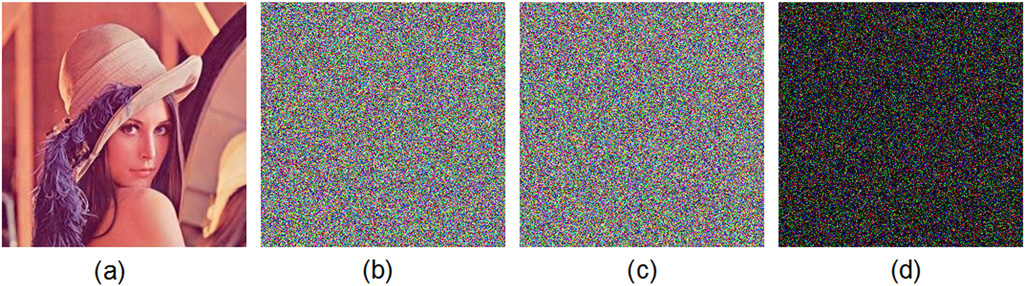
Key sensitivity test I. (a) Original image of Lena, (b) encrypted image of Lena with the secret key K1, (c) encrypted image of Lena with the secret key K2, (d) difference image.

**Fig 12:**
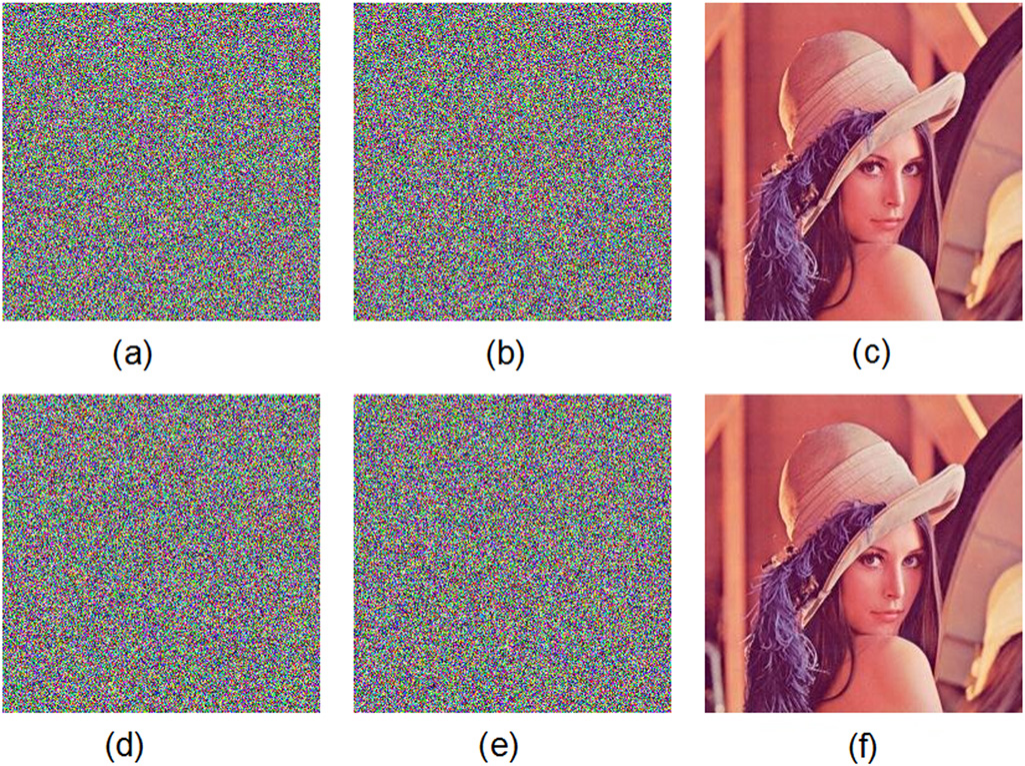
Key sensitivity test II. (a) Encrypted image of Lena with the secret key K1, (b) decrypted image with the secret key K2, (c) decrypted image with the secret key K1, (d) encrypted image of Lena with the secret key K2, (e) decrypted image with the secret key K1, (f) decrypted image with the secret K2.

The original color image of Lena is firstly encrypted by using the secret key K1 and then encrypted by using the secret key K2. We get two ciphered images by two slightly different keys. Fig. 11 displays the test results. The test shows that there is a difference up to 99.64% in terms of pixel gray-scale values between the encrypted image with K1 (Fig. 11(b)) and the encrypted one with K2 (Fig. 11(c)). Moreover, in Fig. 12, we have shown the results of some attempts to decrypt an encrypted image with slightly different secret keys. We use the color image of Lena as the plain-image. Fig. 12(a) shows the encrypted image by using the secret key K1. Fig. 12(b) displays the decrypted image by using another trivially modified key K2. Fig. 12(c) plots the decrypted image by using the correct key K1. The encrypted image by using the secret key K2 is displayed in Fig. 12(d). The decrypted image by using the slightly different key K1 is shown in Fig. 12(e). The decrypted image by using the correct key K2 is depicted in Fig. 12(f). Obviously, the decryption with a slightly different key fails completely and hence the proposed image encryption scheme is highly key sensitive.

**Fig 13:**
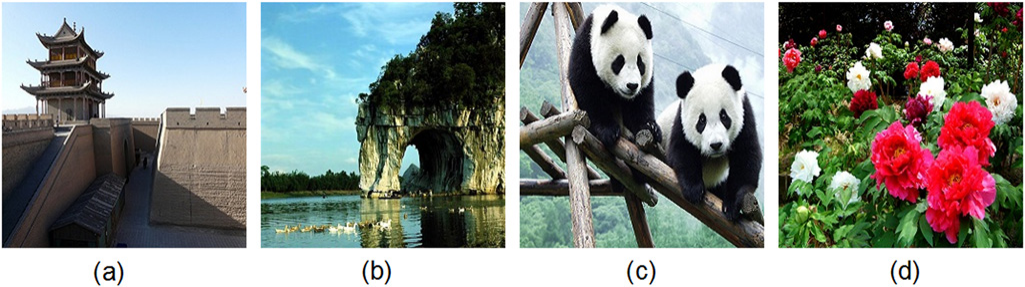
Some original plain-images used in Table 6. (a) City gate tower, (b) Elephantine mountain, (c) Pandas, (d) Peony.

Furthermore, as discussed in Section 0.3, the transformations used in Equations (5)–(15) are constructed such that the initial conditions and parameters of the CML and the fractional-order Chen chaotic system are highly sensitive to a slight change even in one bit of the secret key, which will lead to undesired decryption images. The average pixel differences of some color images (Figs. 5(a), 6(a) and 13) using the random keys K1 and K2 are given in Table 6. From the results in Table 6, one can easily see that the values obtained by the proposed method are very close to the expected value of pixel difference on two randomly generated images (NPCR = 99.6094% and UACI = 33.4635%).

**Table 6:** Comparison of pixel difference between images encrypted by keys with one-bit difference.

Image	NPCR (%)			UACI (%)		
	Red	Green	Blue	Red	Green	Blue
Lena	99.591	99.593	99.629	33.349	33.51	33.561
Vegetables	99.637	99.611	99.605	33.396	33.549	33.583
City gate tower	99.608	99.644	99.583	33.429	33.537	33.340
Elephantine mountain	99.632	99.562	99.628	33.260	33.450	33.349
Pandas	99.634	99.634	99.615	33.498	33.529	33.530
Peony	99.614	99.623	99.628	33.462	33.505	33.522

### 1.6 Speed performance

Besides the security consideration, the running speed of the algorithm is also an important issue for a well applicable cryptosystem, especially for real-time Internet applications. We implement the proposed algorithm by using Matlab 7.1. The speed performance is tested in a computer with an Intel Core 2 Duo CPU 2GHZ, 1.99GB Memory and 600GB hard-disk capacity, and the operating system is Microsoft Windows XP. From Refs. [47, 48, 49], we know that the encryption time of Refs. [5, 50, 51] are 3.704s, >10s and 2.901s, respectively. Table 7 shows the comparison of the experimental results between the proposed encryption method and other image cryptosystems in [5, 50, 51, 52]. Compared to the encryption schemes in [5, 50, 51, 52], we can see that the operation speed of our method is clearly faster for the image of Lena.

**Table 7:** Comparison of encryption time between our proposed method and some other cryptosystems.

Algorithm	Encryption Time (seconds)
The proposed algorithm	1.25
Ref. [5]	3.704
Ref. [50]	>10
Ref. [51]	2.901
Ref. [52]	2.3

## Tolerance of Image Processing

Taking into account the variation tolerance of image processing operations such as noise addition, cropping, JPEG compression etc., the ability of surviving from these attacks for an image encryption scheme is also crucial, apart from the security consideration. *PSNR* (Peak Signal-to-Noise Ratio) is used in this paper to analyze the visual quality of the decrypted image *I*’ in comparison with the plain-image *I*. *PSNR* is defined as:

$$PSNR=\text{20}\log_{\text{10}}\left(\text{255}/\sqrt{MSE}\right)\text{dB,}$$ (37)

where *MSE* is the mean squared error between the plain-image *I* and the cipher-image *I*’, which is given by

$$MSE=\frac{1}{MN}\sum_{i=0}^{M-1}\sum_{j=0}^{N-1}{\left[I(i,j)-I^{\prime }(i,j)\right]}^{2}.$$ (38)

The higher the *PSNR* value is, the less distortion is there to the plain image. Generally speaking, when the value of *PSNR* ⩾ 30, the human eyes cannot percept differences between the plain-image and the decrypted image. When no attack occurs, the *PSNR* value of the decrypted image (Fig. 5(c)) is 76.28. If we observe the original plain-image (Fig. 5(a)) and the decrypted image (Fig. 5(c)), we can not find any visual degradation.

In the following, several common image processing operations such as noise addition, cropping, JPEG compression, rotation, brightening and darkening are performed on our proposed encryption algorithm. Results for the “Lena” image (Fig. 5(a)) are shown in this section. Table 8 displays the *PSNR* values of the decrypted image as the cipher-image is attacked by different image processing operations. The results demonstrate that the decrypted image is still recognizable despite the cipher-image being seriously distorted. The attacks are described as follows.

**Table 8:** The *PSNR* of decrypted image under different image processing operations.

Image processing operations	*PSNR* (dB)
Pepper & Salt Noise 0.005	47.57
Pepper & Salt Noise 0.05	31.87
Pepper & Salt Noise 0.5	19.11
Gaussian Noise [0, 0.05]	19.25
Cropping 25%	29.15
Cropping 50%	23.88
Compression (Quality Factor = 80)	18.63
Compression (Quality Factor = 50)	18.65
Rotation 45°	18.65
Brighten	18.68
Darken	18.66

### 2.1 Noise addition

**Fig 14:**
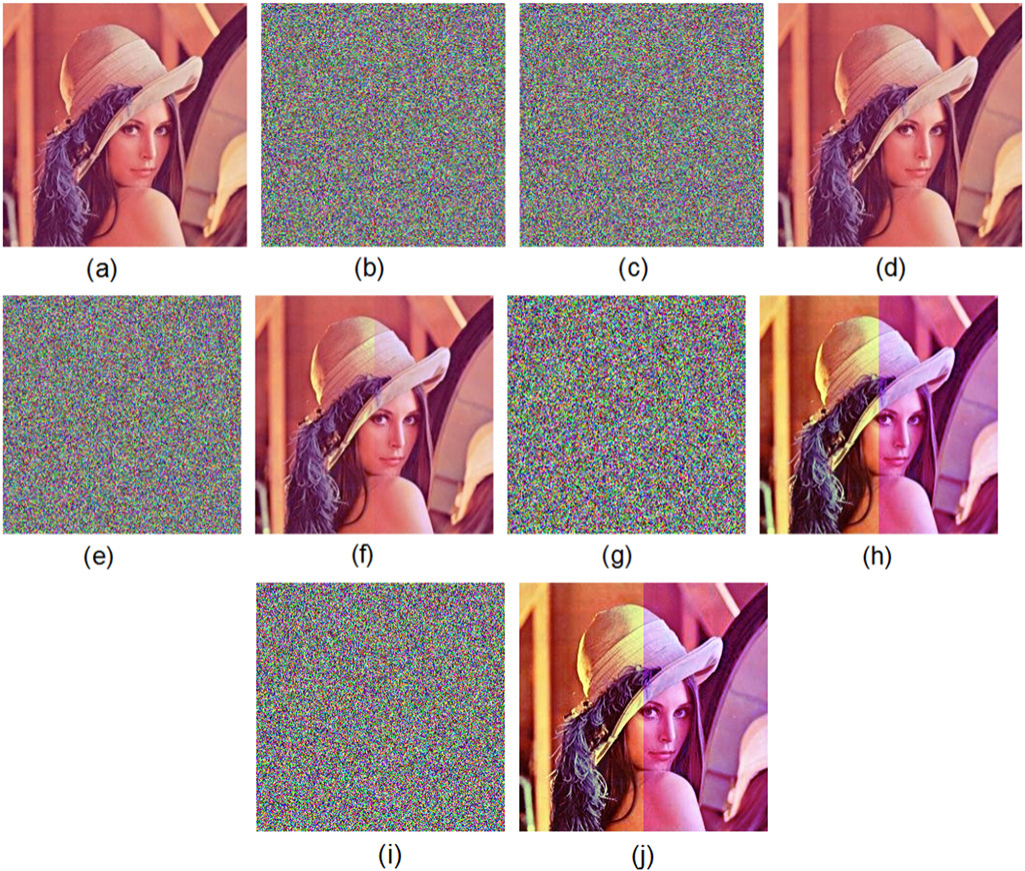
Test of noise addition. (a) Original image of Lena, (b) encrypted image of Lena without noise addition, (c) encrypted image of Lena under adding Pepper & Salt noise with noise density 0.005, (d) decrypted image under Pepper & Salt noise addition (noise density 0.005), (e) encrypted image of Lena under adding Pepper & Salt noise with noise density 0.05, (f) decrypted image under Pepper & Salt noise addition (noise density 0.05), (g) encrypted image of Lena under adding Pepper & Salt noise with noise density 0.5, (h) decrypted image under Pepper & Salt noise addition (noise density 0.5), (i) encrypted image of Lena under Gaussian white noise addition, (j) decrypted image under Gaussian white noise addition.

Generally, addition of noise is responsible for the degradation and distortion of the image. The cipher-image is also degraded by noise addition, resulting in difficulties in image decryption. We tested the proposed scheme’s robustness against two types of noise: Pepper & Salt noise and Gaussian noise, which are added to the cipher-image. Fig. 14(a) and (b) show the plain-image of Lena and the corresponding cipher-image without noise addition, respectively. We add Pepper & Salt noise with different noise densities, i.e., 0.005, 0.05 and 0.5, to the cipher-image, as displayed in Fig. 14(c), (e) and (g), respectively. The proposed scheme is utilized to decrypt the noise-contaminated ciphered images. The decrypted images are shown in Fig. 14(d), (f) and (h), respectively. The corresponding *PSNR* values of the decrypted images are 47.57dB, 31.87dB and 19.11dB, respectively. In addition, Gaussian white noise with mean value 0 and variance value 0.05 is added to the cipher-image, as shown in Fig. 14(i). Fig. 14(j) plots the decrypted image of the cipher-image in Fig. 14(i). Here the *PSNR* value of the decrypted image is 19.25dB. The results demonstrate that the noise-added encrypted image can still be decrypted appropriately, i.e., most information can be recovered.

### 2.2 Cropping

**Fig 15:**
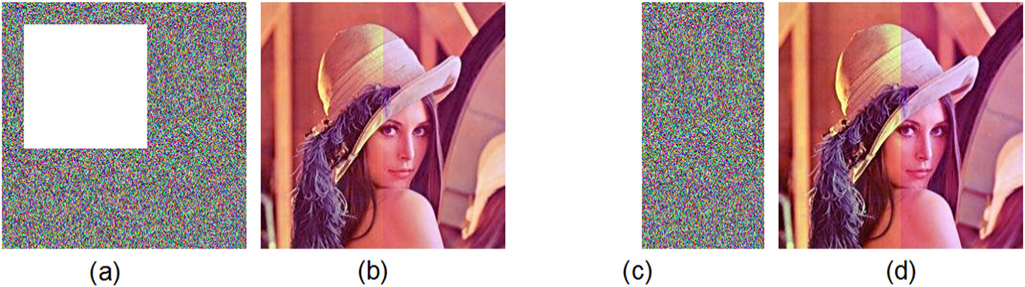
Test of image under cropping. (a) Cropped cipher-mage by removing 25% of the encrypted image of Lena (Fig. 14(b)), (b) decrypted image of the cropped cipher-image (a), (c) cropped cipher-mage by removing 50% of the encrypted image of Lena (Fig. 14(b)), (d) decrypted image of the cropped cipher-image (c).

Image cropping is very common in real applications. Cropping removes the outer parts of an image to enhance framing, accentuate subject matter or modify aspect ratio, which is a lossy manipulation. Fig. 15(a) shows that 25% of the cipher-image is removed where 255 is inserted to the cropped pixels, and then the decrypted image is well obtained using the proposed scheme (Fig. 15(b)). The corresponding *PSNR* value is 29.15. Even there were only a half of the encrypted image remained (Fig. 15(c)), the deciphered image is still recognizable, as shown in Fig. 15(d). Here the *PSNR* value is 23.88. In fact, we can always decrypt the cropped cipher-image with most recover information when the cropped part has a size of less than 256×256 pixels.

### 2.3 JPEG compression

**Fig 16:**
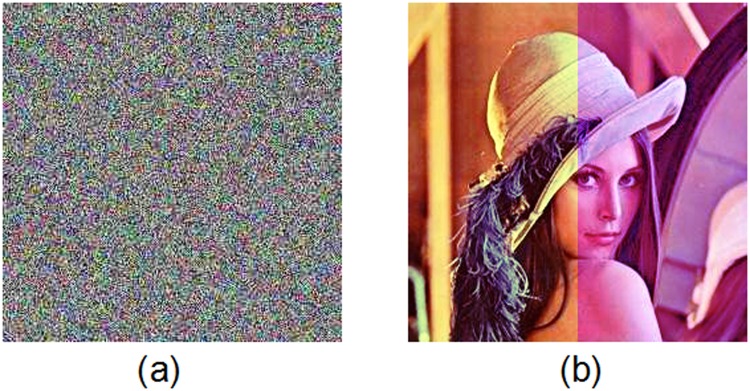
Test of image encryption with JPEG compression. (a) JPEG compressed cipher-mage of Lena, (b) decrypted image under JPEG compression.

Image compression is another very prevalent operation in digital images. In testing the tolerance of JPEG compression, the results show that the encrypted image, if being JPEG compressed, can be decrypted by the designed image encryption scheme, and the decrypted image after JPEG compression is still recognizable. A test is shown in Fig. 16. Fig. 16(a) displays the cipher-image after JPEG compression, where the quality factor used by the JPEG compression is 50. Here, the quality factor is a kind of measure for JPEG compression, commonly within a range between 1 to 100: the bigger the factor, the better the quality of the image after JPEG compression and, correspondingly, the smaller the compression rate. The *PSNR* value of the decrypted image (Fig. 16(b)) is 18.65dB. Further simulation results show that the deciphered image can still be decrypted with most recover information while any quality factor in [1,100].

### 2.4 Rotation

**Fig 17:**
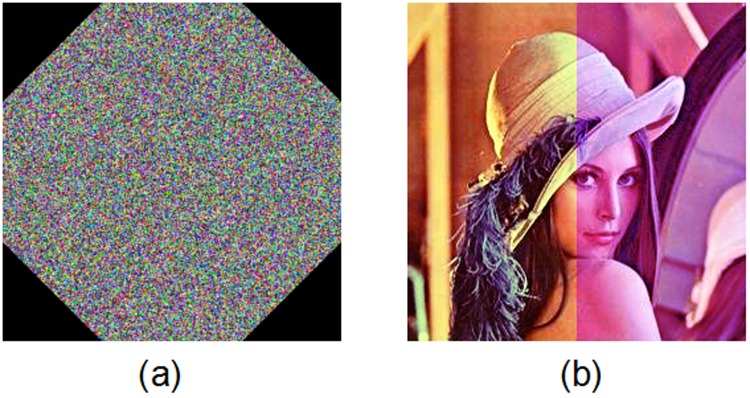
Test of image under rotation. (a) Rotated cipher-image of Lena, (b) decrypted image under rotation.

Image rotation makes the coordinate axes changed. Without synchronization of the orthogonal axes, one cannot decrypt the cipher-image correctly. Here, we do not consider the question of how to recover the axes, which have been geometrically distorted. We assume that the distorted axes have been recovered before the cipher-image is decrypted. Simulations have shown that in this case we can still decipher the encrypted image (Fig. 17(b)) when the ciphered image is rotated by 45°, as shown in Fig. 17(a). Here the value of PSNR is 18.65dB.

### 2.5 Brightening and darkening

**Fig 18:**
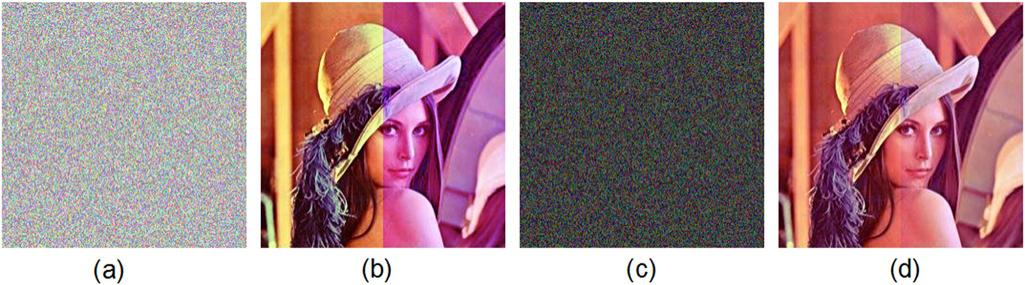
Test of image under brightening and darkening. (a) Brightened cipher-image of Lena, (b) decrypted image under brightening, (c) darkened cipher-image of Lena, (d) decrypted image under darkening.

We also test the proposed scheme’s robustness against image brightening and darkening attacks, which increases or decreases the color intensities in a colormap. Simulation results are given in Fig. 18. Fig. 18(a) and (c) display the cipher-image after brightened and darkened, respectively. Fig. 18(b) and (d) show the deciphered images decrypted from the brightened and darkened encrypted images, respectively, where the PSNR values of the decrypted images separately are 18.68dB and 18.66dB. These test results show that the ciphered image after brightened or darkened can still be decrypted with most recovered information.

**Remark 5**. From the above results, one can easily conclude that the proposed image encryption technique is highly robust against some common image processing operations and geometric attacks, e.g. noise addition, cropping, JPEG compression, rotation, brightening and darkening.

## Conclusions

In this paper, we have proposed a new color image encryption algorithm based on a CML and a fractional-order chaotic system. The presented cryptosystem is composed of four processes, i.e., an image division-shuffling process, a key streams generation process, an image permutation process and an image diffusion process, to enhance the security and sensitivity of the cryptosystem. Moreover, the generation of the key streams and the image division-shuffling process are carried out simultaneously in a parallel mode, which accelerate the operation speed of our method. Experimental results have demonstrated that, comparing with current image encryption algorithms, the proposed encryption algorithm has a better performance in terms of security, sensitivity, speed and robustness. Furthermore, corresponding results also show that the presented encryption method efficiently overcomes the drawbacks in the present one-dimensional chaotic image encryption algorithms.
